# Prevalence of hypermobility in children and adolescents aged 10–13 years in correlation with postural defects—a preliminary report

**DOI:** 10.3389/fpubh.2026.1856830

**Published:** 2026-09-08

**Authors:** Klaudia Paula Czorniej, Wojciech Kułak

**Affiliations:** Department of Paediatric Neurology and Rehabilitation, Faculty of Health Sciences, Medical University of Białystok, Białystok, Poland

**Keywords:** adolescents, Beighton scale, Brighton criteria, children, joint hypermobility, postural defects

## Abstract

**Introduction:**

Joint hypermobility, defined as excessive joint range of motion, may be asymptomatic or associated with musculoskeletal complaints and postural abnormalities. Benign Joint Hypermobility Syndrome (BJHS) is diagnosed according to the Brighton criteria. The aim of the study was to determine the prevalence of hypermobility among Polish children aged 10–13 years and its association with postural defects and pain complaints.

**Materials and methods:**

The study included 100 children (51% girls, 49% boys). The Beighton Scale, Brighton criteria, postural assessment, and knee and foot tests were used. Data were analyzed using *χ*^2^ tests, *Z*-tests, Kruskal–Wallis test, Spearman correlation, with *p* < 0.05 considered statistically significant.

**Results:**

Joint hypermobility was identified in 35% of participants, more frequently in girls. Full-symptom BJHS was rare (<5%), whereas 39% of children met major or minor Brighton criteria. Excessive joint mobility was correlated with mild postural abnormalities (*ρ* = 0.21–0.35; *p* < 0.05). In multivariable logistic regression adjusted for age and sex, the association between Beighton score and clinical signs suggestive of scoliosis was no longer statistically significant (aOR = 1.25; 95% CI: 0.91–1.70; *p* = 0.164). Children with higher Beighton scores more often reported pain and joint instability.

**Conclusion:**

Joint hypermobility is present in a significant proportion of children, more commonly in girls, and is associated with mild postural deformities and musculoskeletal complaints. Early diagnosis and individualized preventive strategies may reduce the risk of chronic pain development and progression of postural defects.

## Introduction

Joint hypermobility is defined as a range of motion exceeding normal physiological limits and represents a clinical feature rather than a disease itself. It may occur as an isolated finding or as part of heritable connective tissue disorders, including hypermobile Ehlers–Danlos syndrome (hEDS) and Hypermobility Spectrum Disorders (HSD) (1–8). The Beighton score is the most widely used screening tool for generalized joint hypermobility, while the Brighton criteria, despite being largely replaced in clinical practice by the 2017 hEDS/HSD classification, continue to be used in epidemiological studies to facilitate comparison with previous research (6–8).

Joint hypermobility is common in childhood, with reported prevalence varying from approximately 10 to 25% depending on age, sex, ethnicity, diagnostic criteria, and study population (9–14). It is generally more frequent in girls than in boys and tends to decrease with age (11–14). Although many children remain asymptomatic, generalized joint hypermobility has been associated with musculoskeletal pain, joint instability, and selected postural abnormalities, including increased lumbar lordosis, genu valgum, flat feet, and spinal asymmetry (2, 15–23). However, these associations remain inconsistent across studies, and their clinical significance has not yet been fully established.

Despite growing international interest in pediatric joint hypermobility, epidemiological data from Poland remain scarce, particularly regarding its relationship with postural abnormalities in school-aged children (24). Furthermore, most available studies have been conducted in selected clinical populations or have focused primarily on the prevalence of hypermobility rather than its potential musculoskeletal consequences.

This study was intentionally designed as a preliminary cross-sectional investigation. Before undertaking a large multicenter epidemiological study, it was necessary to assess the feasibility of recruitment, the applicability of the assessment protocol, the distribution of Beighton scores, and the frequency of postural abnormalities in Polish schoolchildren. Since epidemiological data on joint hypermobility in Polish children are scarce, the present study was intended to provide initial estimates that could support future hypothesis-driven studies with larger representative samples and multivariable statistical analyses.

Based on the available literature, we hypothesized that joint hypermobility would be relatively common among children aged 10–13 years, occur more frequently in girls, and be associated with selected musculoskeletal complaints and postural abnormalities. Accordingly, the aims of the present study were to determine the prevalence of joint hypermobility among children aged 10–13 years from Białystok, Poland, to assess sex differences in its occurrence, and to explore its associations with pain complaints and selected postural characteristics, including spinal asymmetry, lumbar lordosis, genu valgum, and foot posture.

## Materials and methods

### Participants

The study included 100 children and adolescents aged 10–13 years. The final sample consisted of 55 girls (55%) and 45 boys (45%). The mean age of participants was 11.26 years (range: 10–13 years). Most participants (83%) were between 10 and 12 years of age.

Initially, 200 children were screened for eligibility. Participants who did not meet the predefined age criterion (10–13 years) or did not fulfill the inclusion criteria were excluded from further analysis. The final study sample consisted of 100 children.

Participants were recruited from several schools in Białystok and from children referred for rehabilitation assessment at the participating rehabilitation center. Recruitment was based on participant availability and parental consent. Therefore, the study sample should be considered a convenience sample rather than a representative sample of the entire pediatric population of Białystok or Poland.

All participants belonged to the White population, reflecting the demographic characteristics of the local recruitment area.

A detailed sociodemographic characterization of the respondents is presented in Table 1.

**Table 1:** Sociodemographic characteristics of the respondents.

Gender	*N*	%
Girls	55	55%
Boys	45	45%
Age		
10 years	32	32%
11 years	27	27%
12 years	24	24%
13 years	17	17%
Ethnicity (Race)		
White	100	100%

### Study design and data collection

A cross-sectional study was conducted at the Paediatric Rehabilitation Clinic within the Early Assistance Centre for Children with Disabilities “Dać Szansę,” in cooperation with the VITA MED Medical Centre in Białystok, Poland.

A total of 100 children and adolescents aged 10–13 years were enrolled in the study. Prior to participation, both participants and their parents were informed about the study objectives, procedures, scope of data collection, and data storage methods. Parents received an information sheet regarding their child’s participation. The study was conducted anonymously, and participation was voluntary.

Inclusion criteria were as follows:

- informed consent from both the participant and the parent/legal guardian,
- age between 10 and 13 years.

Exclusion criteria included:

- lack of consent to participate in the study,
- age outside the 10–13-year range,
- medical conditions preventing participation in physical assessment.

The study was conducted using a questionnaire and observational method. Each participant completed:

- a self-administered questionnaire covering the most commonly reported musculoskeletal complaints.

The questionnaire contained simple age-appropriate questions concerning musculoskeletal symptoms, including pain, fatigue, recurrent sprains, and perceived joint instability. Participants completed the questionnaire under supervision of the investigator and were allowed to request clarification whenever necessary to ensure correct understanding of the terminology used.

- In addition, a physical examination was performed, including:
  – the modified Beighton Scale (Beighton score) for the assessment of joint hypermobility,
- visual postural assessment,
- Bertrand Adams test (Adam’s forward bend test),
- measurement of knee valgus and varus using a measuring tape (Knee alignment was assessed through visual examination and measurement of intermalleolar and intercondylar distances using a measuring tape).

Assessment of spinal alignment included visual postural examination, the Adams forward bend test, and scoliometer measurements. In a small proportion of participants, radiographic examinations had been previously performed as part of routine clinical care; however, radiographic findings were not systematically available for all participants and were therefore not included in the study analyses. Consequently, the term “scoliosis” in this study refers to clinical findings suggestive of spinal asymmetry rather than radiologically confirmed scoliosis.

Lumbar lordosis and other postural characteristics were evaluated through standardized visual postural assessment performed in the standing position. Increased lumbar lordosis was defined clinically as an excessive inward curvature of the lumbar spine identified during visual examination in the standing position.

Postural abnormalities were identified when deviations from accepted anatomical alignment were observed during clinical examination. Assessment included evaluation of head position, shoulder symmetry, spinal curvatures, pelvic alignment, lower-limb alignment, and foot posture.

The applied tools enabled the assessment of joint hypermobility and its relationship with postural abnormalities.

Foot morphology was evaluated through visual assessment of the longitudinal arch and by the heel-rise (toe-standing) test, which was used to assess flexibility of the foot arch.

All physical examinations were performed by Klaudia Paula Czorniej, MSc, physiotherapist, using a standardized assessment protocol. To improve assessment reliability, all findings were subsequently reviewed by Wojciech Kułak, Professor of Pediatric Neurology and Rehabilitation.

The examination protocol was standardized before study initiation. All participants were assessed according to the same clinical procedures and examination sequence to minimize measurement variability.

### Scales

The assessment of joint hypermobility commonly uses the modified Beighton score. It comprises five clinical tests, four of which are assessed bilaterally. The maximum possible score is 9 points, with 1 point awarded for each positive test result (25, 26).

A score of 4 points or higher indicates the presence of joint hypermobility (22). Joint range of motion was assessed using a standard universal goniometer. All Beighton score measurements were performed according to standardized procedures described in the literature. Hyperextension angles of the elbow and knee joints were confirmed by goniometric measurements rather than visual estimation alone.

The diagnostic criteria for joint hypermobility using the modified Beighton score are presented in Table 2.

**Table 2:** Individual tests and scoring system in the Modified Beighton score.

Assessed side	Right	Left
Elbow joint hyperextension > 10°	1	1
Passive apposition of the thumb to the forearm	1	1
Hyperextension of the metacarpophalangeal (MCP) joints > 90°	1	1
Knee joint hyperextension > 10°	1	1
Placing the palms flat on the floor during forward flexion with the knees fully extended	1	

Although contemporary classifications increasingly emphasize Hypermobile Ehlers–Danlos Syndrome (hEDS) and Hypermobility Spectrum Disorders (HSD), the Brighton criteria remain widely used in epidemiological and screening studies. Therefore, they were applied in the present study to facilitate comparison with previous literature. The Brighton criteria include both the modified Beighton score and the presence of selected clinical manifestations associated with generalized joint hypermobility. They include both the modified Beighton score, which assesses the range of joint mobility, and the presence of selected clinical features. The most important of these include joint pain, predisposition to hernias, cutaneous and ophthalmological changes, joint subluxations, Marfanoid body habitus features, varicose veins, degenerative changes of the spine, and pelvic organ prolapse, such as uterine or rectal prolapse. These manifestations are divided into major and minor criteria, and the diagnosis of BJHS is based on specific combinations of these criteria (Table 3) (22, 27).

**Table 3:** Brighton criteria.

Major criteria	Beighton score ≥4/9 (current or historical) Arthralgia affecting ≥4 joints, lasting longer than 3 months
Minor criteria	Beighton score 1–3/9 Pain in 1–3 joints or lumbosacral region lasting >3 months; degenerative changes of the spine or spondylolisthesis Dislocation or subluxation in >1 joint or recurrent instability in a single joint Periarticular soft tissue tenderness Marfanoid habitus Skin abnormalities (stretch marks, hyperextensibility, “paper-like” scars) Ptosis, myopia, ocular features (e.g., oblique palpebral fissures, opposite to Down syndrome pattern) Varicose veins, hernias, uterine or rectal prolapse
BJHS is diagnosed when the following criteria are met: two major criteria, or one major criterion and two minor criteria, or four minor criteria, or two minor criteria, provided that the patient is a first-degree relative of a person diagnosed with BJHS.	

Current evidence indicates that the Beighton score is a reliable clinical tool characterized by high and, in some cases, very high inter-rater reliability, regardless of the examiner’s level of experience. Importantly, it can also be applied in patients with intellectual disability (28, 29).

In patients suspected of BJHS who present with pain and joint swelling, differential diagnosis for inflammatory or autoimmune diseases is necessary. For this purpose, laboratory testing is recommended, including complete blood count (CBC), antinuclear antibodies (ANA), erythrocyte sedimentation rate (ESR), rheumatoid factor, and immunoglobulin levels (IgG, IgA, IgM) (22).

### Procedure and ethical considerations

The study was conducted in accordance with applicable guidelines and was reviewed and approved by the Bioethics Committee of the Medical University of Białystok (resolution no. APK.002.6.2024).

### Statistical analysis

Data were processed using Microsoft Excel 2013 and analyzed using Statistica PL software version 13.0 (StatSoft, Poland).

Descriptive statistics were calculated for all study variables. Continuous variables were presented as means ± standard deviations or medians with interquartile ranges, depending on data distribution, whereas categorical variables were expressed as frequencies and percentages. The normality of distribution was assessed using the Shapiro–Wilk test. Between-group comparisons were performed using the *χ*^2^ test or Fisher’s exact test for categorical variables and the Mann–Whitney U test or Kruskal–Wallis test for continuous variables, as appropriate. Spearman’s rank correlation coefficient was used to evaluate associations between Beighton scores and selected postural abnormalities.

To determine whether generalized joint hypermobility was independently associated with postural abnormalities, multivariable logistic regression analyses were additionally performed. Separate models were constructed for each outcome variable, with age and sex included as potential confounding variables. Adjusted odds ratios (aORs) with 95% confidence intervals (95% CI) were calculated. A two-sided *p*-value < 0.05 was considered statistically significant.

## Results

The number of girls and boys in the study sample was relatively comparable—girls accounted for 55% of the sample (55 participants), while boys accounted for 45% (45 participants). Statistical analysis (chi-square test) showed that this difference was not statistically significant (*p* > 0.05), indicating that the sex distribution in the sample can be considered relatively balanced. The balanced representation of both sexes allows for comparisons between groups with adequate sample sizes.

In the assessment of body posture, most participants demonstrated either normal posture or only minor deviations. Correct head and shoulder girdle alignment was observed in 75% of participants, while forward head posture was found in 35%. Normal chest configuration was observed in 80% of participants, whereas abnormalities were present in 20% (Z = 4.23; *p* < 0.001). Normal abdominal alignment was found in 65% of participants, while deviations were present in 35% (Z = 3.11; *p* = 0.002). Regarding spinal curvatures, normal thoracic kyphosis was observed in 65% of participants, while increased kyphosis was present in 35%.

Normal lumbar lordosis was present in 75% of participants, whereas its deepening was observed in 25% (*χ*^2^ = 10.57; df = 2; *p* = 0.005). A spine without scoliosis was found in 65% of participants, whereas signs suggestive of scoliosis, identified through postural assessment, Adams forward bend test, and scoliometer examination, were observed in 35% of participants, with severe curvature occurring sporadically (*p* < 0.001). Normal alignment of the lower limbs was observed in 65% of participants, while abnormalities were present in 35% (*χ*^2^ = 9.34; df = 1; *p* = 0.002). Normal foot arch was present in 55% of participants, flattening in 35%, and flat-valgus foot was identified in 10% of participants (*χ*^2^ = 8.76; df = 2; *p* = 0.013) (Table 4).

**Table 4:** Postural and spinal assessment in the study population.

Assessment item	Normal/minimal deviation (%)	Abnormal (%)
Head and shoulder alignment	75	25
Chest	80	20
Abdomen	65	35
Thoracic kyphosis	65	35
Lumbar lordosis	75	25
Scoliosis	65	35
Knee alignment	65	35
Foot arch	55	35

The results of the Bertrand–Adams test showed no abnormalities of the trunk in 70% of the examined participants, the presence of a paraspinal muscle prominence in 25%, and a rib hump in 5% of participants (*χ*^2^ = 18.21; df = 2; *p* < 0.001). Spearman’s correlation demonstrated a moderate positive relationship between the presence of a paraspinal muscle prominence and scoliosis (*ρ* = 0.42; *p* < 0.01) (Table 5).

**Table 5:** Bertrand–Adams test—assessment of trunk asymmetry.

Change	Percentage of participants (%)
No changes	70
Paraspinal muscle prominence	25
Rib hump	5

The assessment of joint hypermobility using the modified Beighton score showed that most participants did not present generalized joint hypermobility. A score of 0 points was observed in 25% of children, 1–3 points in 40%, while a score indicative of hypermobility (≥4 points) was found in 35% of participants (4–5 points: 16%, 6–7 points: 8%, 8–9 points: 11%).

The distribution of Beighton scores was non-normal (Shapiro–Wilk test: W = 0.92; *p* < 0.001).

The Kruskal–Wallis test demonstrated statistically significant differences in Beighton scores between upper and lower limb components of the scale (H = 12.7; *p* = 0.005).

Spearman’s correlation analysis revealed a weak but statistically significant association between Beighton score and the presence of clinical signs suggestive of mild scoliosis (*ρ* = 0.21; *p* = 0.04) (Table 6).

**Table 6:** Beighton scale.

Score (points)	Number of individuals	% of population	Interpretation
0	25	25	No features of joint hypermobility
1–3	40	40	No or minimal joint hypermobility
4–5	16	16	Moderate joint hypermobility
6–7	8	8	Marked joint hypermobility
8–9	11	11	Generalized joint hypermobility

The analysis of the Brighton criteria showed that full-symptom joint hypermobility syndrome was rare (<5%). No criteria were met in 63% of participants, major criteria were present in 19%, minor criteria in 20%, while combinations of major and minor criteria occurred sporadically (*χ*^2^ = 27.43; df = 3; *p* < 0.001). Combined criteria were observed only occasionally. Extended analysis demonstrated a significant association between hypermobility and postural deformities (*ρ* = 0.35; *p* < 0.01). Multivariable logistic regression analysis adjusted for age and sex showed that Beighton score was not independently associated with clinical signs suggestive of scoliosis (aOR = 1.25, 95% CI: 0.91–1.70; *p* = 0.164). Age (aOR = 1.42, 95% CI: 0.93–2.18; *p* = 0.107) and sex (female vs. male: aOR = 0.91, 95% CI: 0.36–2.31; *p* = 0.848) were also not significantly associated with the outcome (Table 7).

**Table 7:** Multivariable logistic regression analysis of factors associated with clinical signs suggestive of scoliosis.

Variable	Adjusted OR (aOR)	95% CI	*p*-value
Beighton score	1.25	0.91–1.70	0.164
Age (years)	1.42	0.93–2.18	0.107
Female sex	0.91	0.36–2.31	0.848

aOR, adjusted odds ratio; CI, confidence interval. The model was adjusted for age and sex.

The Mann–Whitney U test revealed significant differences in Beighton scores between participants with and without paraspinal muscle prominence (U = 234; *p* = 0.01) (Table 8).

**Table 8:** Brighton criteria—joint hypermobility syndrome.

Criteria	Number of participants	% of population
No criteria met	63	63
Major criteria	19	19
Minor criteria	20	20
Combination: major + minor criteria	Rare	–

## Discussion

The present preliminary study demonstrated that joint hypermobility is a relatively common finding among children aged 10–13 years and occurs more frequently in girls than in boys. Children with greater joint mobility more often presented mild postural abnormalities, including spinal asymmetry, increased lumbar lordosis, genu valgum, and flat feet, and they more frequently reported musculoskeletal complaints such as pain and joint instability. Although full-symptom benign joint hypermobility syndrome was uncommon, many participants exhibited selected clinical features associated with hypermobility. These findings suggest that increased joint mobility may be associated with early musculoskeletal and postural changes in univariate analyses. However, after adjustment for age and sex, these associations were no longer statistically significant, indicating that demographic factors may partly explain the observed relationships.

The broad issue of joint hypermobility has in recent years attracted considerable interest among researchers (30). These studies primarily focus on the prevalence of joint hypermobility, associated conditions, and the search for effective therapeutic approaches (30–32). The literature reports that this condition affects between 0.6% and as many as 31.5% of patients, depending on the applied criteria, such as age, sex, or race (33). Differences between sexes and ethnic groups are also observed. In women, joint hypermobility occurs approximately 1.5–3 times more frequently than in men, while in Asian and African populations it is significantly more common than in Caucasian populations (33–35).

No data regarding the prevalence of joint hypermobility among Polish children were found in the literature. According to American data, hypermobility affects 12% of children aged 5–17 years, with a prevalence of 18% in girls and 7% in boys (36). In Greek children of a similar age range, prevalence was reported at 8.8% (37), whereas in children aged 9–13 years it may reach 25% (38). These differences are likely due to inconsistencies in diagnostic criteria and variability in study populations (39). The present study showed that joint hypermobility in children aged 10–13 years occurred in 25% of participants, which is consistent with data reported for Western populations (approximately 10–25%) (9, 24). However, these findings should be interpreted with caution because the study sample was recruited from a single rehabilitation setting and cooperating schools and therefore may not be representative of the entire pediatric population of Białystok or Poland. The higher prevalence in girls confirms previous findings indicating that hypermobility is more common in females (14), supporting the hypothesis that sex is a significant predictor of joint hypermobility.

Adjustment for age and sex was performed because both variables are known to influence the prevalence of generalized joint hypermobility during childhood. Although univariate analyses suggested relationships between hypermobility and selected postural abnormalities, these associations were attenuated after multivariable adjustment. This finding suggests that demographic characteristics may partly explain the observed differences and highlights the importance of considering potential confounding variables in future studies.

In the Polish literature, diagnosis of hypermobility in children is often based on tests developed by Sachse, modified by Kapandji (31, 40). Mikołajczyk et al. (41) identified hypermobility in 10 girls (8.3% of the studied group) using a criterion of 7 positive clinical tests out of 13, while no boys met this criterion. Hypermobile children frequently reported lower limb pain associated with fatigue. Smolewska et al. (42) indicate that musculoskeletal pain is a common reason for medical consultation in children and may suggest hypermobility after excluding inflammatory joint diseases (41).

In English-language literature, the most commonly used diagnostic tool is the modified Beighton score, developed based on the Brighton criteria (43, 44). This scale includes a set of clinical tests assessing joint mobility of major upper and lower limb joints and the ability to perform specific hypermobility movements. A score of at least 4 out of 5 tests is considered indicative of hypermobility (39, 45, 46). In addition, alternative assessment methods such as Carter, Bulbena, Marshall, and Wilkinson tests are used; some, such as the Marshall test, focus solely on thumb opposition to the forearm (39, 45, 46).

Diagnosis of joint hypermobility according to the Brighton criteria is based on both the Beighton score and clinical manifestations (22). Major criteria include a Beighton score ≥4 and joint pain in at least four joints lasting more than 3 months. Minor criteria include lower Beighton scores, musculoskeletal pain persisting for at least 3 months, recurrent dislocations or subluxations, periarticular soft tissue symptoms, Marfanoid features, skin abnormalities (e.g., stretch marks, hyperextensibility, thin or “papyraceous” scars), ocular abnormalities, and varicose veins, hernias, or pelvic organ prolapse (22, 41). Assessment using the Brighton criteria showed that full-symptom BJHS was rare (<5%), while a considerable proportion of participants met isolated major or minor criteria (39%), consistent with previous studies (22, 27).

Despite varying cutoff values in pediatric populations, the Beighton score remains the primary screening tool for hypermobility (44, 45). Gedalia et al. (36) and Lamari et al. (47) used a cutoff of 3/9 in British children, while Smith-Engelsman et al. (44) applied a threshold of 5/9 in Dutch populations. In the present study, a threshold of ≥4 points indicated hypermobility, observed in 35% of children.

Differences in prevalence may result from variability in diagnostic criteria and from the fact that Beighton-based systems include both complications and phenotypic features of heritable connective tissue disorders (19). Remvig et al. (12) emphasize that postural or ocular features may be of limited diagnostic value in BJHS. After exclusion of connective tissue diseases, BJHS diagnosis remains possible (19, 48). Again, BJHS was found to be rare (<5%), while isolated criteria were present in 39% of participants.

Although most participants demonstrated generally correct posture, minor deviations such as forward head posture and mild lower limb deformities were observed in some children. These findings are consistent with the literature suggesting that joint hypermobility may predispose to subtle postural abnormalities, including genu valgum, flat feet, increased lumbar lordosis, and mild spinal curvature disorders (22, 23). The observed weak association between hypermobility severity and spinal changes suggests that increased joint laxity may be one contributing factor to postural development, although other factors are also involved. Although statistically significant, the observed correlations were weak (*ρ* = 0.21–0.35) and therefore should not be interpreted as evidence of strong clinical relationships.

Postural defects were identified in the majority of children, suggesting a relationship between hypermobility and musculoskeletal abnormalities. Berwecki et al. (49) demonstrated that chronic fatigue, low physical activity, and reduced muscle strength may contribute to postural defects; 36% of hypermobile patients had additional postural abnormalities, most commonly scoliosis (19%). In the present study, mild scoliosis was observed in 35% of children, highlighting the need for rehabilitation and genetic assessment. Spearman’s rank correlation demonstrated a weak but statistically significant association between hypermobility and clinical signs suggestive of scoliosis. However, this association was not confirmed in the multivariable logistic regression model after adjustment for age and sex.

Pain complaints, joint instability, and paraspinal muscle prominence further confirm that hypermobility is associated with musculoskeletal symptoms, including back and limb pain, subluxations, and increased susceptibility to soft tissue injury (21, 22). Significant differences in Beighton scores between participants with and without paraspinal muscle prominence suggest a possible compensatory muscular mechanism, consistent with symptomatic hypermobility described in the literature (10, 22).

Symptoms of hypermobility may include pain, impaired proprioception in small and knee joints, reduced muscle reflexes, and degenerative changes (41, 50–55). In the present study, 8.9% of children reported pain during excessive physical activity, while no joint swelling was observed. Back pain occurred in 11.1% of participants.

In summary, the findings indicate that joint hypermobility was observed relatively frequently in this sample of children aged 10–13 years from Białystok, was more common in girls, and was associated with selected postural abnormalities and musculoskeletal complaints. Early recognition enables preventive and physiotherapeutic interventions that may reduce the development of chronic pain and postural disorders (3, 10, 22).

Rehabilitation should be individually tailored, focusing on strengthening periarticular muscles and improving proprioception (41, 56, 57). Appropriately prescribed therapeutic exercise has been shown to reduce pain and improve function (58–60). However, physical activity was not assessed in the present study and should be included in future investigations to better understand its relationship with joint hypermobility and postural abnormalities. Individualized therapy remains essential to prevent future complications (41, 61–65).

Further epidemiological studies in Poland involving larger and more representative pediatric populations are needed to better define the prevalence and clinical significance of joint hypermobility. Future studies should also include assessment of physical activity and other lifestyle factors, as well as longitudinal follow-up, to better understand their influence on musculoskeletal development and postural abnormalities (14, 24).

Despite these limitations, the study provides valuable data and may serve as a basis for further research using more objective methods such as video analysis, digital goniometry, or 3D scanning.

## Conclusion

1. In this sample of children aged 10–13 years from schools of Białystok, joint hypermobility was observed in approximately one-quarter of participants and was more common in girls than in boys.
2. Higher Beighton scores were associated with selected postural abnormalities in univariate analyses. However, these associations were not confirmed as independent after adjustment for age and sex. Therefore, the findings should be interpreted as exploratory and require confirmation in larger prospective studies.
3. The observed associations between hypermobility and postural characteristics were statistically significant but generally weak, and therefore should be interpreted with caution.
4. Full-symptom BJHS was uncommon, whereas isolated major or minor Brighton criteria were observed more frequently.

## Study limitations

The study has several limitations. First, posture assessment was based mainly on visual observation and simple clinical tests, which may have affected measurement accuracy and reproducibility. Although a scoliometer and goniometer were used when appropriate, most postural variables remained clinically assessed, and radiographic confirmation of spinal deformities was not available for all participants or included in the analyses. Second, despite the use of standardized tools, including the Beighton score and Bertrand–Adams test, assessment of features such as muscle prominence or subtle asymmetries may still have been partly subjective. Third, the cross-sectional design precludes conclusions about causality or changes over time. Fourth, as this was a preliminary study, a relatively small convenience sample of 100 children recruited from schools in Białystok was included. Consequently, the findings demonstrate associations rather than causal relationships and cannot be generalized to the entire Polish pediatric population. Finally, in the multivariable logistic regression analyses, adjustment was performed only for age and sex, as these are well-established determinants of generalized joint hypermobility in children and were the only relevant confounding variables available in the dataset. However, information on body mass index, pubertal status, and habitual physical activity was not available and therefore could not be included in the statistical models. In addition, other lifestyle, ergonomic, and environmental factors were not fully quantified or controlled. Furthermore, the study did not include a control group. Consequently, residual confounding cannot be excluded, and the observed associations should be interpreted with caution. Future studies should include larger, more representative samples together with comprehensive assessment of anthropometric, developmental, behavioral, and environmental factors to better evaluate the independent relationships between joint hypermobility and postural abnormalities.

## Data Availability

The raw data supporting the conclusions of this article will be made available by the authors, without undue reservation.
